# Writing Behavior of Phospholipids in Polymer Pen Lithography (PPL) for Bioactive Micropatterns

**DOI:** 10.3390/polym11050891

**Published:** 2019-05-15

**Authors:** Alessandro Angelin, Uwe Bog, Ravi Kumar, Christof M. Niemeyer, Michael Hirtz

**Affiliations:** 1 Institute of Biological Interfaces (IBG-1), Karlsruhe Institute of Technology (KIT), Hermann-von-Helmholtz-Platz 1, 76344 Eggenstein-Leopoldshafen, Germany; alessandro.angelin@ntu.edu.sg (A.A.); niemeyer@kit.edu (C.M.N.); 2 Institute of Nanotechnology (INT) & Karlsruhe Nano Micro Facility (KNMF), Karlsruhe Institute of Technology (KIT), Hermann-von-Helmholtz-Platz 1, 76344 Eggenstein-Leopoldshafen, Germany; uwe.bog@kit.edu (U.B.); ravi.kumar@kit.edu (R.K.)

**Keywords:** polymer pen lithography, PPL, phospholipids, biomimetic membranes, supported lipid membranes, supported lipid bilayers, scanning probe lithography

## Abstract

Lipid-based membranes play crucial roles in regulating the interface between cells and their external environment, the communication within cells, and cellular sensing. To study these important processes, various lipid-based artificial membrane models have been developed in recent years and, indeed, large-area arrays of supported lipid bilayers suit the needs of many of these studies remarkably well. Here, the direct-write scanning probe lithography technique called polymer pen lithography (PPL) was used as a tool for the creation of lipid micropatterns over large areas via polymer-stamp-mediated transfer of lipid-containing inks onto glass substrates. In order to better understand and control the lipid transfer in PPL, we conducted a systematic study of the influence of dwell time (i.e., duration of contact between tip and sample), humidity, and printing pressure on the outcome of PPL with phospholipids and discuss results in comparison to the more often studied dip-pen nanolithography with phospholipids. This is the first systematic study in phospholipid printing with PPL. Biocompatibility of the obtained substrates with up to two different ink compositions was demonstrated. The patterns are suitable to serve as a platform for mast cell activation experiments.

## 1. Introduction

Supported lipid bilayers (SLBs) have attracted much interest for decades now as model membranes for studying processes in cell membranes and for biosensing applications [1], but also—especially in recent years—as interfaces to living cells in biological and biomedical experiments [2]. While many experiments and applications can utilize uniform, large-area SLBs that are most often prepared by simple vesicle fusion approaches, more sophisticated setups can be realized by structured SLBs. The methods used to obtain these are manifold, ranging from combination of top-down and bottom-up approaches, e.g., electron beam lithography or photolithography-generated structures in combination with vesicle fusion, to soft-lithography methods [3] and direct-write scanning probe lithography (SPL) [4,5,6].

Polymer pen lithography (PPL) [7] is a SPL method of particular interest here, as many applications in cell biology require the functionalization of quite large surface areas (~cm^2^) while at the same time multiplexed (i.e., more than one molecular compound or “color” within a written pattern) features of sub-cellular scale are needed. PPL is a hybrid technology combining aspects of microcontact printing (µCP) and dip-pen nanolithography (DPN) [8]. It utilizes a polymer stamp (usually made of polydimethylsiloxane (PDMS)) bearing tens of thousands of tips on a square centimeter-sized area [9] that can all print on a surface in parallel, thus speeding up the patterning process massively [10]. It offers material integration similar to µCP but has a highly controlled patterning process that allows for arbitrary patterns by multiple approaches to the surface that can be precisely moved in lateral directions at the same time (instead having the desired pattern predefined on the stamp itself) and multiplexing [5,11,12,13]. Usually, PDMS stamp-based approaches, like µCP, are used to print pre-patterns, altering surface chemistry for subsequent self-assembly of SLBs in the fluid phase. However, direct transfer prints of lipids from such stamps to the surface were also reported [5,14,15,16]. In particular, multiplexed lipid patterns of 1,2-dioleoyl-sn-glycero-3-phosphocholine (DOPC) admixed with different fluorophore-modified phospholipids on a micron scale over large surface areas (~cm^2^) printed by PPL were demonstrated by Brinkmann et al. for potential application as a biointerface [5]. In a similar approach, it was also shown that PPL can generate different types of lipid patterns, forming a gradient over mm scale distances, while maintaining subcellular sizes of the single features within the printed pattern [16]. However, a systematic study of the influence of PPL process parameters when patterning phospholipids is still lacking. Elucidating the influence of typical PPL process parameters (as dwell time, environmental humidity, and printing pressure) would allow for a better and more rational approach in generating phospholipid micro patterns.

Here, we conducted the first systematic study of the influence of dwell time (i.e., duration of contact between tip and sample), humidity, and printing pressure on the outcome of PPL with phospholipids. The results are compared with PPL of other ink systems and with DPN with phospholipids. Finally, the printing results are tested for feasibility in biological applications by a mast cell activation demonstration experiment. 

## 2. Materials and Methods

### 2.1. Materials

**Figure 1:**
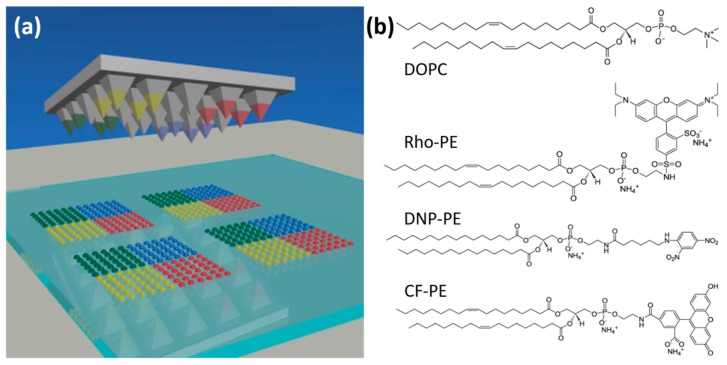
Scheme of polymer pen lithography (PPL) and the phospholipids used in the present study. (**a**) A polymer stamp featuring pyramidal pens is inked with different phospholipids in distinct segments and then used for printing of multiplexed lipid arrays. (**b**) The chemical structure and abbreviated names for the different phospholipids used in the printing experiments.

The phospholipids used in the study were obtained in chloroform (Avanti Polar Lipids, Alabaster, AL, USA) and used as received. The phospholipids in use were 1,2-dioleoyl-sn-glycero-3-phosphocholine (DOPC), 1,2-dioleoyl-sn-glycero-3-phosphoethanolamine- N-(lissamine rhodamine B sulfonyl) (Rho-PE), 1,2-dipalmitoyl-sn-glycero-3-phosphoethanolamine-N-[6-[(2,4-dinitrophenyl)amino]hexanoyl] (DNP-PE), and 1,2-dioleoyl-sn-glycero-3-phosphoethanolamine-N-(carboxyfluorescein) (CF-PE). Chemical structures are shown in Figure 1b.

For biological antibody binding and cell recruiting experiments, primary Alexa Fluor® 647 (AF647)-labeled anti-DNP IgE antibody was obtained by labelling anti-DNP IgE antibodies (D8406, Sigma Aldrich, Steinheim, Germany) with an Alexa Fluor® 647 Antibody Labeling Kit (A20186, ThermoFisher, Dreieich, Germany). In addition, secondary goat anti-mouse IgG (H+L) Highly Cross-Adsorbed Secondary Antibody (A32728, ThermoFisher, Dreieich, Germany) was used.

### 2.2. Preparation of Masters, Stamps, Inks, and Substrates

A 4-inch Silicon oxide wafer was vapor-coated with Bis(trimethylsilyl)amine (HDMS) and spin-coated with the positive photoresist AZ 1505 at 1500 rpm for 60 s. After a 5 min prebake at 95 °C, the photoresist was exposed to UV light with a dosage of 80 mJ/cm^2^, using a shadow mask that was designed to allow the illumination of a 50 × 50 array of squares with a pitch of 100 µm. After the UV exposure, the wafer was developed with AZ 726 for 30 s, followed by a postbake at 90 °C for 30 min. Afterwards, the wafer was etched with hydrofluoric acid (BHF) for 20 min to remove the photoresist-free silicon oxide. Then, the photoresist was stripped with AK 400K, and the silicon was etched through the square oxide-free windows with 30% potassium hydroxide (KOH) at 70 °C for 60 min. Since this wet etching process is anisotropic, pyramidal well structures were formed. Finally, the residual silicon oxide was stripped with BHF for 60 min. The obtained master surface was modified with trichloro(1H,1H,2H,2H-perfluorooctyl)silane by gas phase silanization to avoid elastomer adhesion during PDMS stamp fabrication. To this aim, the silicon masters were sonicated in chloroform (for 5 min), in ethanol (for 5 min), and finally dried with compressed air. Subsequently, the masters were treated with oxygen plasma (0.2 mbar, 300 W, 100% O_2_) for 5 min and incubated in a desiccator under vacuum in presence of 100 μL of trichloro(1H,1H,2H,2H-perfluorooctyl)silane.

The pen array stamps were prepared with hard PDMS (hPDMS). For the PDMS polymerization reaction, four components are simultaneously required [10]. Three of these components were pre-mixed in a stock solution by combining 3.4 g of (7–8% vinylmethylsiloxane)-dimethylsiloxane (AB116647, abcr, Karlsruhe, Germany), 18 μL of 2,4,6,8-tetramethyl-2,4,6,8-tetravinylcyclotetrasiloxane (396281, Sigma Aldrich, Steinheim, Germany), and one drop of platinum-divinyltetramethyldisiloxane (catalyst, AB153234, abcr, Karlsruhe, Germany). The mixture was stirred and subsequently degassed in a vacuum desiccator for 30 min and stored at 4 °C.

Prior to PDMS molding, the silicon masters were sonicated in chloroform (for 5 min), in ethanol (for 5 min), and finally dried with compressed air. Directly before use, 1 g (25–35% Methylhydrosiloxane)-dimethylsiloxane copolymer (AB109380, abcr, Karlsruhe, Germany) was added to 3.4 g of the pre-mixed hPDMS solution, stirred, promptly degassed, and immediately poured onto the pre-cleaned silicon masters. To facilitate the further handling, the poured hPDMS was sandwiched between the silicon master and a pre-cut microscopy glass slide, which was carefully placed and gently pressed against the silicon master to allow a homogeneous hPDMS distribution and to remove eventual trapped air bubbles. The PDMS polymer was cured at 72 °C overnight, allowed to cool down at room temperature, and stored at room temperature until use.

For the preparation of the ink containing lipids, a carrier lipid DOPC was mixed with either functionalized or fluorophore-conjugated lipids (depending on the experiment) and diluted with chloroform to a final lipid concentration of 10 mM. For one-color experiments, DOPC was mixed with 1 mol% Rho-PE. For two-color experiments, DOPC was mixed with either 1 mol% Rho-PE or 2 mol% FITC-PE (the higher concentration of FITC-PE allows to compensate the lower brightness and stability of the FITC fluorophores). For the cell recruiting experiments, 10 mol% of DNP-PE was added to the Rho-PE-doped ink solution.

The inks were subsequently used for the PDMS stamp’s inking as described in the following.

Before use, the excess of PDMS around the silicon master was carefully removed by a scalpel, and the PDMS stamp was detached from the master. Subsequently, the PDMS stamp was treated with oxygen plasma (0.2 mbar, 300 W, 100% O_2_) for 2 min to render the surface hydrophilic. Finally, 4 μL of a 10 mM ink solution were used in the case of a 5 × 5 mm^2^ stamp, and the spin-coating was performed at 6000 rpm for 30 s.

Microscopy cover glasses (18 × 18 mm) were used as substrates after cleaning by subsequent sonication in chloroform (for 5 min), isopropanol (for 5 min), and H_2_O (3 times for 5 min). Finally, the substrates were dried under a nitrogen stream and stored at room temperature.

### 2.3. Polymer Pen Lithography (PPL) Procedure

For the PPL process, a self-built setup was used. First, the ink-loaded PDMS stamp was mounted at the end of a moveable arm. Stamp positioning in *x* and *y* is realized by stepper motors. The chip substrates were placed on a sample table equipped with goniometer stages, allowing for angular adjustment between stamp and substrate along the *x*- and *y*-axis. By another stepper motor, the table is operated in *z* to establish contact between stamp and substrates.

Prior to the actual PPL process, a sacrificial substrate was placed on the sample holder for stamp levelling. Then, levelling was performed by visual observation of the pens through the backside of the PPL stamp while establishing contact with the substrate, using a microscope equipped with a CMOS camera. When in contact with the substrate, the soft PDMS pens deform. Supporting images of not contacted and contacted pyramids and the levelling procedure are shown in Figure S1.

A non-planar parallel orientation between the stamp and the substrate results in non-uniform deformation of the pens along the stamp. By comparison of the images from the four stamps’ corners while in contact, the amplitude and direction of the misalignment can be estimated. The two goniometer stages are then utilized to compensate for this misalignment. In practice, the substrate is lowered to remove the physical contact between stamp and substrate, the motorized goniometers are moved, and finally the correction is checked by re-establishing the contact between stamp and substrate. The described approaching/imaging/tilting process is cyclically repeated until an equal contact force of all pyramids in the four stamps’ edges is achieved. 

After the planar parallel alignment had been obtained, the actual printing procedure was performed. The stages were controlled by a PS90 motion controller (OWIS), and coordinated motion sequences for the PDMS stamp stages were created in the on-board software OWISsoft (OWIS, Staufen im Breisgau, Germany). The movements of the goniometers, the illumination, and camera zoom were controlled by a custom software user interface. These software packages also allow to control the dwell time (contact time of stamp to substrates within the printing sequence) and stamp holder extension (for controlled varying of stamp sample distance, as used in the trial of printing pressure dependence, Section 3.5). By extending the stamp further than necessary to first touch a sample, the applied printing pressure is raised, thus the controlled extension can be used as proxy for the (for lack of pressure sensor) not directly accessible printing pressure [17]. The humidity in the printing environment was controlled by a self-built climate control system consisting of a plexiglass chamber and a feedback system that can feed moist or dry nitrogen into the system depending on the measured relative humidity (RH). By this, a stable RH controlled to ±0.5% (in the range up to 50% RH) and ±1.0% (above 50% RH) could be achieved in the sample printing chamber (Figure S2).

### 2.4. Optical Microscopy

Fluorescence images were recorded by Axiovert 200M microscope (Zeiss, Oberkochen, Germany) using the software Axio Vision 4.7 (Zeiss, Oberkochen, Germany). The microscope was equipped with the following filters: Cy3: Exc = BP 550/25 (HE), Em = BP 605/70 (HE), Cy5: Exc = BP 640/30, Em = BP 690/50, eGFP: Exc = BP 475/40, Em = BP 530/50.

### 2.5. Data Evaluation and Analysis

Images of the substrates were recorded by fluorescence microscopy and analyzed with the software ImageJ [18,19]. The recorded images had a resolution of 0.6 μm/pixel, and all measurements were rounded to the nearest whole number. For the analysis of area and intensity of the spots, the images were first rotated by 5–7° (to allow the correct numbering of the recognized particles, see below) and subsequently duplicated to create a mask by intensity thresholding. Note that the thresholds were manually set for each image. For comparison of different images, the exposure time and threshold were maintained equal. Finally, the area containing a single array was selected on the mask image, the function "analyze particles" was used to automatically identify the printed spots, and the area and intensity values were read from the original image. Note that the scale was set to measure the distances/areas in μm. The obtained values were further analyzed in the software Excel (Microsoft).

### 2.6. Biological Demonstration Experiment

Before printing the substrates used in the biological experiments, the excess of ink loaded on the stamp was reduced by printing sacrificial substrates with long dwell times (10 s), firstly, to enable lower numbers of lipid layers, and secondly, to avoid diffusion of lipids onto unprinted areas during the first washing/blocking with aqueous buffers. This method was preferred to loading less ink onto the stamp to avoid unequal ink distribution. The glass substrates for biological experiments were then printed at a relative humidity (RH) of 40% and with fixed dwell and pause times.

The glass substrates bearing arrays of DNP-conjugated lipids for antibody binding and cell recruiting experiments were incubated with 500 μL of blocking solution (10 mg/ml BSA in PBS -/-) for 10 min and with 500 μL of Tyrode’s buffer (137 mM NaCl, 2.7 mM KCl, 1 mM MgCl_2_, 1.8 mM CaCl_2_, 0.2 mM Na_2_HPO_4_, 12 mM NaHCO_3_, 5.5 mM glucose, 0.1% BSA) for 10 min, respectively.

In the case of antibody binding experiments, the substrates were incubated with 50 μL of a 1:50 dilution of an Alexa Fluor® 647 (AF647)-labeled anti-DNP IgE antibody for 30 min and promptly rinsed 3 times with Tyrode’s buffer. Subsequently, the substrate was incubated with 50 μL of a 1:50 dilution of a secondary Alexa Fluor® 647 (AF647)-labeled goat anti-mouse IgG antibody for 30 min, promptly rinsed 3 times with Tyrode’s buffer, and imaged by fluorescence microscopy.

In the case of cell recruiting experiments, mast cells RBL 2H3 were detached from the culture flask by treatment with trypsin and counted with a Neubauer chamber. Cells were diluted to a final concentration of 400 cells/μL in RBL 2H3 cells medium (70% MEM (ATCC, Manassas, WV, USA), 20% RPMI medium 1640 (Gibco-ThermoFisher, Dreieich, Germany), 10% FBS), and incubated with 1:1000 dilution of AF647-labeled anti-DNP IgE antibody for 2 h at 37 °C. Subsequently, the cells were centrifuged at 1500 g for 5 min, the supernatant was removed, and the cells resuspended in Tyrode’s buffer to a final concentration of 1000 cells/μL. The blocked substrates were pre-heated for 30 min at 37 °C and incubated with 100 μL of the obtained cell suspension. Live imaging of the recruiting cells was performed by fluorescence microscopy. After seeding, the cells were incubated at 37 °C for at least 30 min and subsequently imaged. The presented images show recruitment state after 40–45 min.

## 3. Results and Discussions

### 3.1. Printing Phospholipids with PPL

**Figure 2:**
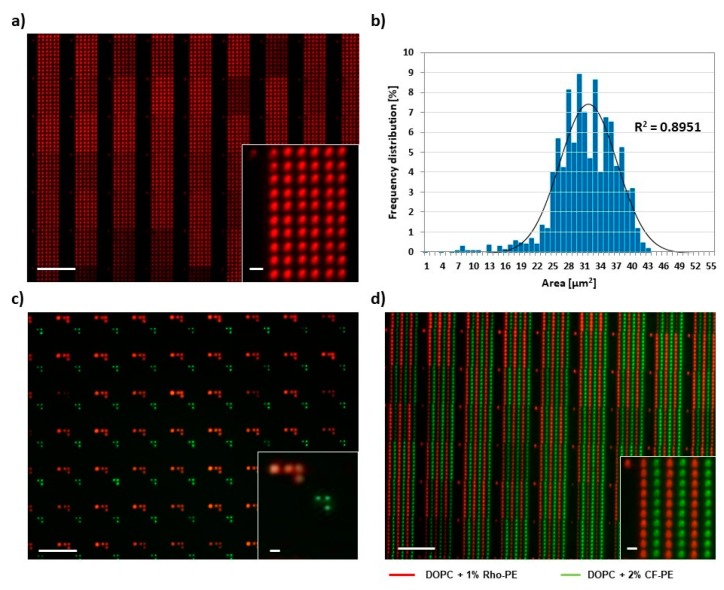
PPL-printed phospholipid arrays. (**a**) Representative fluorescence micrograph of a 6 × 10 spot array of a 1,2-dioleoyl-sn-glycero-3-phosphocholine (DOPC)/Rho-PE mixture printed on glass; (**b**) Histogram of the area distribution of 30 lipid arrays as seen in (**a**); (**c**) Example for arbitrary patterning with two inks. Printed inks were DOPC admixed with either 1 mol% Rho-PE (red channel) or 2 mol% CF-PE (green channel). Details on the alignment challenge and offset correction in two-color printing are shown in Figure S3; (**d**) Example of a multiplexed phospholipid array (same inks as in (**c**). The obtained two-color substrate was further analyzed by measuring the distances between spots within and among the different ink patterns. As shown in Figure S4, the measured distances are in accordance to the theoretical values, confirming the success of alignment and printing procedure. The scale bars in all panels equal 100 µm in the main image and 10 µm in the insets.

To ensure printing worked as expected prior to systematic studies, mono and multiplexed printing procedures were trialed. For this, dye-doped lipid inks were spin-coated onto plasma-treated stamps. After mounting and alignment of the stamp on the self-built printing setup, test prints were conducted at controlled humidity and fixed dwell times. Typical outcomes are presented in Figure 2.

Figure 2a features a regular 6 × 10 spot array printed at 40% RH and room temperature, with a dwell time of 1 s and an in-between spot pause of 4 s. The overall printing result is homogeneous with slight variation of fluorescence intensity from the array blocks printed by different tips of the stamp (mean intensity per block 62254 ± 14839 with *n* = 30) A Gaussian fit to the feature size distribution gives a mean feature area of (31 ± 6) µm^2^ (Figure 2b), which corresponds to a calculated feature radius (assuming circular shape) of (3.1 ± 0.3) µm. As a further test of the stability of the system, multiplex printing with two differently labelled lipid inks was implemented. After careful alignment of the stamp and calibration for offsets (Figure 2c, Figures S3 and S4), controlled multiplexed patterns could be achieved (Figure 2d).

### 3.2. Influence of Dwell Time

**Figure 3:**
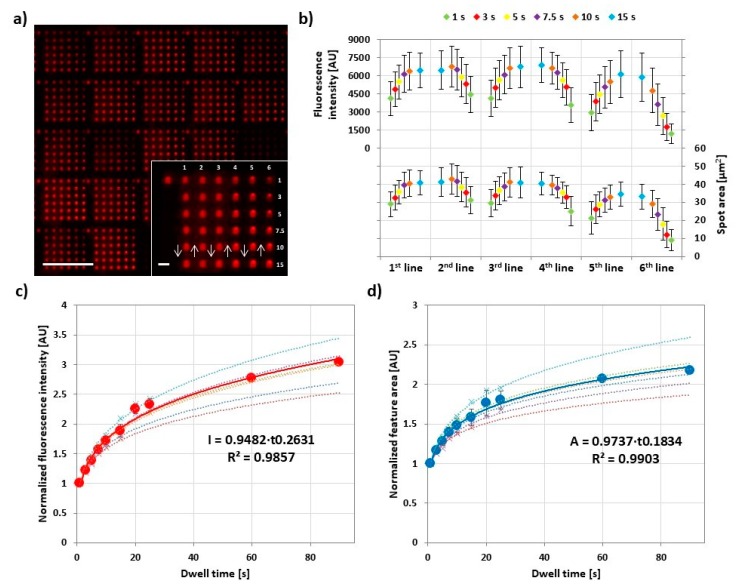
Influence of dwell time on lipid ink transfer. (**a**) Representative fluorescence micrograph of the 6 × 6 arrays designed to analyze the amount of transferred lipid in dependence of dwell time. The inset shows higher magnification of a single array, along with the printing scheme. The scale bars equal 100 µm in the main panel and 10 µm in the inset; (**b**) Plots of the average intensity (above) and area (below) of corresponding spots (same line and same dwell time) in 30 analyzed arrays against the dwell time per line; (**c**,**d**) show plots of the normalized average intensity (red) and area (blue), respectively, of spots against dwell time combined from six independent experiments (data were normalized to the 1 s dwell time value, the original data without normalization are shown in Figure S5). The background plots (connected by dashed curves) show the normalized data from the single experiments for reproducibility evaluation. All the printings were performed under 40% relative humidity (RH) and room temperature. The reproducibility of the printed substrates was proofed by comparing the average areas and intensities among the six independent experiments, as shown in Figure S6. Error bars represent standard deviation.

In L-DPN, feature size strongly depends on the dwell time and—in the case of line writing—on tip speed [4,20,21]. For PPL, feature sizes can be generally dependent on dwell time, but also change with contact pressure (cf. Section 3.5) [7,16,17]. Here, we probed the influence of dwell time in PPL with phospholipids for the DPN-like mode (Figure 3).

To assess the dwell time influence, 6 × 6 feature arrays were printed, while increasing and decreasing dwell time per feature in each column to simultaneously check for hysteresis effects. After printing, for each experiment, 30 arrays were analyzed by fluorescence microscopy. All the prints were performed at 40% RH and room temperature. Lateral feature area was directly measured, while fluorescence intensity was plotted additionally as proxy for feature height [22]. The plot of the feature area and fluorescence intensity averaged for 30 different arrays over the course of an array print (Figure 3b) shows that both parameters rise and fall repeatedly for the first four lines of the print, before ink depletion effect leads to lower intensity and area. At the same time, it becomes clear that overall intensity and area are not extensively depending on dwell time as the intrinsic variance between arrays printed by different tips (expressed in the error bar range) has almost the same influence on overall intensity. The reproducibility of the printed substrates was also proofed by comparing the average areas and intensities among the six independent experiments (Figure S6). The normalized and combined data from the experiments (Figure 3c,d) reveal a common trend for feature area and intensity. Both are growing with dwell time but level off more and more for longer dwell times.

### 3.3. Influence of Humidity during Printing

Feature sizes in DPN and PPL (at least in the more DPN-like transfer mode) are usually dependent on RH during printing [23]. This is due to the water meniscus building up upon contact between tip and surface from the ambient humidity. In the case of phospholipids as ink, the RH dependence in L-DPN is additionally amplified, as the ink itself changes properties (phase state and viscosity) by differences in hydration [24].

**Figure 4:**
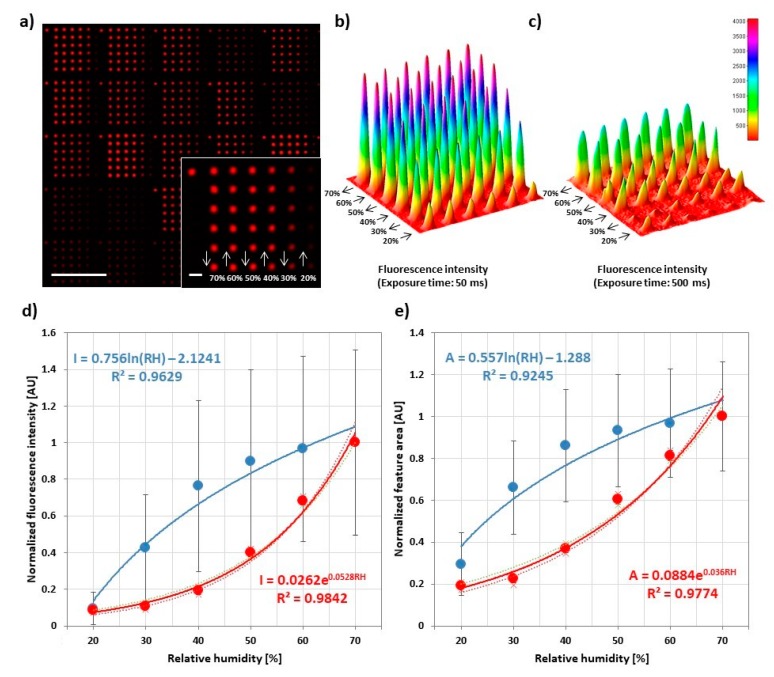
Influence of relative humidity in lipid ink transfer. (**a**) Representative fluorescence micrograph of 6 × 6 spot arrays printed to analyze the amount of transferred lipid in dependence of relative humidity during printing. Dwell time was 1 s for all features. The inset shows higher magnification of a single array along with the printing scheme. The scale bars equal 100 µm in the main image and 10 µm in the inset; (**b**,**c**) Three-dimensional plots of the intensity profile of a single array from two independent experiments; (**d**,**e**) Plots of the average normalized intensity and area of spots against relative humidity for a stamp carrying high ink load (blue) and low ink load (red). Data were normalized on the 70% RH data point to compare the trends from three independent experiments. The analyzed images can be found in Figure S7, along with data before normalization. For each slide, 30 arrays were analyzed. Error bars represent standard deviation.

To probe the influence of RH in PPL with phospholipids, 6 × 6 arrays with different RH in each line were printed. The RH was decreased starting from 70% down to 20% in the last line. After a new RH setpoint was reached (this takes usually less than a minute, refer also to Figure S2), the system was allowed to equilibrate for 5 more min before the start of printing the next features. During equilibration, the lipid ink on the stamp changes in hydration state, leading to an accompanying change in viscosity [4,25], which is known to have a strong impact on ink transfer in DPN and PPL [26,27]. Additionally, a decreasing humidity ramp was chosen to prevent spreading and reformation of features printed at lower humidity when RH is raised, as the lipid patterns are generally sensitive to humidity [14]. Overall, feature intensity and size decrease strongly with decreasing RH (Figure 4). The feature area can be tuned from (43 ± 15) µm^2^ at high humidity to (11 ± 6) µm^2^ at low humidity, corresponding roughly to a calculated feature radius (assuming circular shape) of (3.7 ± 0.7) µm at high humidity to (1.9 ± 0.6) µm at low humidity. This underlines the strong dependence of the printing outcome from RH, hence a need for controlled ambient conditions in regard to humidity. Interestingly, some insights on the ink transfer dynamics can be achieved by a closer inspection of the plots obtained from independent experiments. These were conducted with two different stamps that show high (Figure 4b and blue data points in Figure 4d,e) or low (Figure 4c and red data points in Figure 4d,e) “transfer capability”, being influenced by the amount of ink present on the stamp and/or (in principle) the printing pressure during the experiment. As evident comparing Figure 4b,c, a different transfer capability of the stamps was postulated based on the observed different amount of ink deposited by the stamps despite the same printing parameters (i.e., humidity, dwell time and pause times). A qualitative analysis of the recorded data shows that, beside the deposition of a higher amount of ink, the array in Figure 4b shows a very low depletion effect, only observable at RH < 30%, while the plot in Figure 4c shows equal spots for high RH (>60%) and a relevant depletion effect already for RH < 50%. Hence, at high RH, the high ink fluidity leads to a fast flow of ink from the pyramids’ base and body to the apex during printing and, therefore, to low depletion effects. Contrarily, low RH reduces the ink fluidity, implying a reduced recovery speed and stronger depletion, which can, however, be compensated by the higher transfer capability of the stamp in Figure 4b. These observations allow us to conclude that the higher transfer capability is most likely due to a higher amount of ink present on the stamp rather than the effect of a different printing pressure, since an increased printing pressure would indeed lead to more deposited ink (cf. Section 3.5), but it would not explain the observed differences in depletion. It is also interesting to notice that different trends were observed for the curves obtained with the different stamps—higher absolute intensity values and exponential growth (red data points) in contrast to lower absolute intensity values and a logarithmic growth (blue data points). These effects might be explained by considering the transfer process as the result of a combination of the effects of ink hydration and amount. For high amount of ink load, the influence of RH becomes less pronounced due to an abundance of ink that can rapidly flow from the stamp to the substrate (therefore, a plateau is reached fast upon increasing RH). For lower ink load, the RH becomes the real limiting factor, leading to a more controlled transfer (as also shown by a lower standard deviation). The re-flow of ink from a reservoir at the stamp’s tips’ bases at humidities > 40% also explains why depletion effects are only seen at low humidities, when the amount of ink available on the tip is relevantly reduced by the amount of ink deposited in each feature. At higher humidities, material from the reservoir (which holds a much larger amount of ink, only insignificantly reduced by the volume deposited in a printed feature) can replenish the tip apex easily. Printing of over 100 features without depletion was reported [5], and usually stamps can be used for all samples produced for one experiment without running out of ink (up to 15 substrates were produced with a single stamp, still showing no depletion). Finally, the analysis of the plot of spot area against RH (Figure 4e) reveals the same trends as the corresponding intensities curve. However, it is interesting to notice that the absolute area values share the same range throughout the three experiments, in contrast to the intensity values. Altogether, the results indicate that the ink flow rate increases in relation to the environmental RH and to the ink load on the pyramids, similar to L-DPN [21,24]. That the area is less influenced than intensity by the amount of transferred ink indicates that high ink flow rates tend to create multi-layered (hence higher) spots [22]. 

### 3.4. Combined Effects of Dwell Time and Humidity during Printing

**Figure 5:**
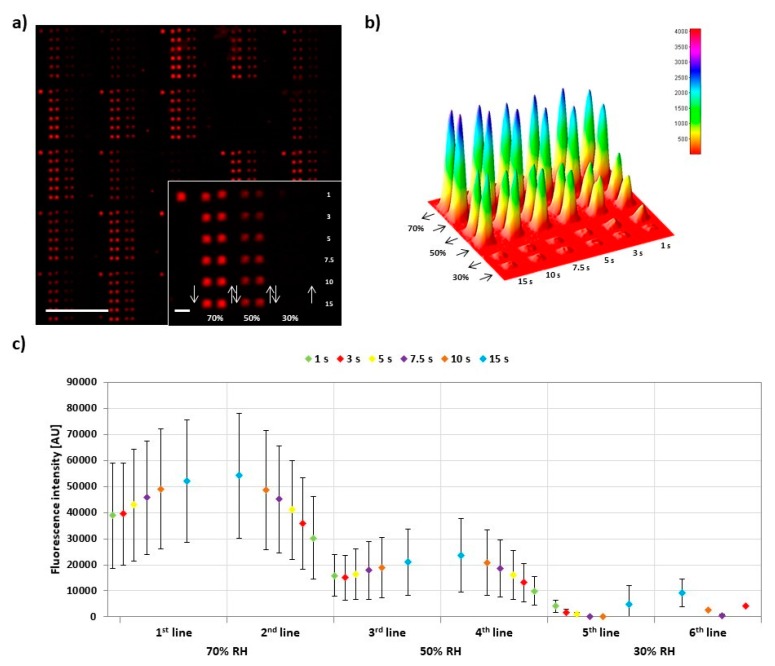
Combined effect of dwell time and humidity on lipid ink transfer. (**a**) Representative fluorescence micrograph of the printed 6 × 6 arrays designed to analyze the amount of transferred lipid in dependence of relative humidity and dwell time. The inset shows higher magnification of a single array along with the printing scheme. The scale bars equal 100 µm in the main image and 10 µm in the inset; (**b**) Three-dimensional plot of the intensity profile of the single array from the inset in panel (**a**); (**c**) Plot of the average intensity of corresponding spots (same line, same RH and same dwell time) in 30 analyzed arrays against the dwell time and grouped by RH. Error bars represent standard deviation.

To further elucidate the tuning possibilities for PPL with phospholipids, a combined ramping of dwell time and humidity was implemented (Figure 5). Here, the combination of both effects discussed in the previous two sections result in a pronounced tuning of feature intensity. When comparing the outcome at different humidity, it can be observed that the variation in feature intensity from different tips (given by the error bars) is—relative to the overall intensity—increasing with decreasing humidity. This implies a more irregular replenishing of lipid ink at the tip apex for lower humidity, compatible with the more viscous state of the lipid ink at low hydration [21].

### 3.5. Effect of Printing Pressure

**Figure 6:**
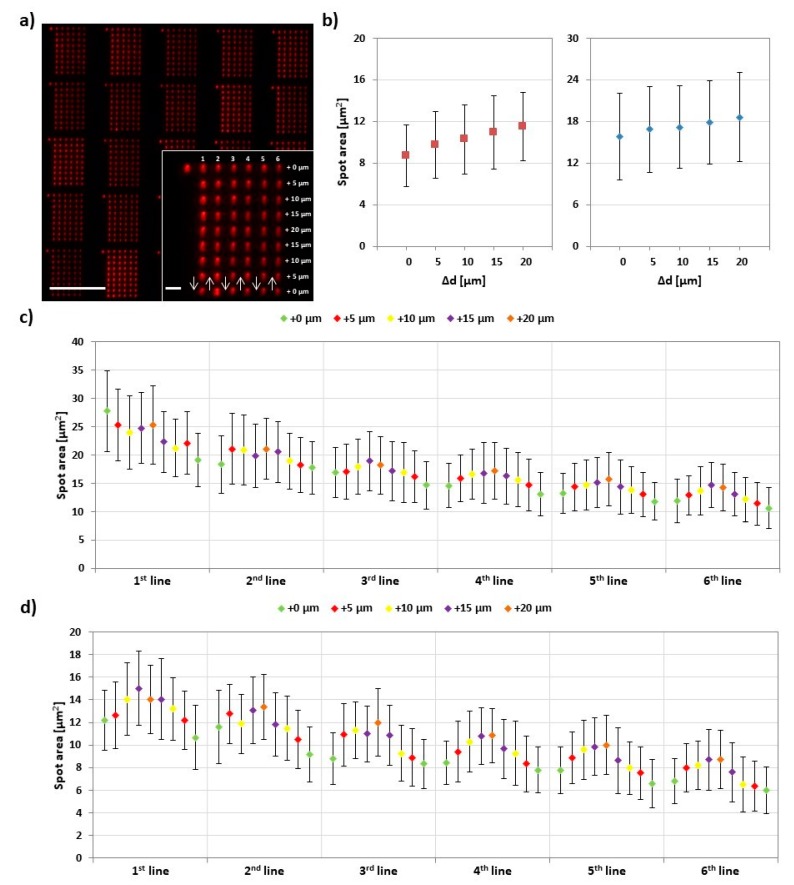
Effect of printing pressure on lipid ink transfer. (**a**) Representative fluorescence micrograph of the printed 9 × 6 arrays designed to analyze the amount of transferred lipid in dependence of relative pressure increase Δd (i.e., increase in stage advancement (d). The inset shows higher magnification of a single array along with the printing scheme. The scale bars equal 100 µm in the main image and 10 µm in the inset; (**b**) Plot of the average area against Δd in two independent experiments. Equivalent plots for the average fluorescence intensities against Δd in the two experiments are shown in Figure S8; (**c**,**d**) Plots of average area of corresponding spots (same line and same Δd) against Δd in 30 analyzed arrays in the two experiments. Error bars represent standard deviation.

In PPL with classical thiol inks (like 16-mercaptohexadecanoic acid (MHA)), the printing pressure has a profound impact on feature size [7,17]. When the flexible PDMS tips of the PPL stamp array are pressed onto the substrate, they deform elastically and contact bigger surface areas with increasing pressure. This results in quite substantial size differences, independent of used dwell time. As the applied pressure or force is not accessible in most printing setups, the difference Δd in the vertical movement between the stage/sample plane and the stamp, relative to the first printed feature, is often used for quantification. Figure 6 presents corresponding results.

Here, 9 × 6 feature arrays were printed with an increasing and decreasing stage movement of additional 5 µm per step during a line. The Figure 6b shows the result of such ramps for two different experiments with slightly different absolute starting positions of the stamp (hence different ranges for the feature sizes during ramping). Here, features’ areas range from (9 ± 3) µm^2^ to (12 ± 3) µm^2^, corresponding roughly to a calculated feature radius (assuming circular shape) of (1.7 ± 0.3) µm to (1.9 ± 0.3) µm, and (16 ± 6) µm^2^ to (19 ± 6) µm^2^, (2.2 ± 0.5) µm to (2.4 ± 0.4) µm, respectively. When looking at the feature size evolution for single lines (Figure 6c,d), it becomes visible that, while the overall trend of feature size increase and decrease over a ramp is consistent, it is overlaid by an ink depletion effect, leading to an overall decrease of average size. This effect is more pronounced here than in the dwell time experiments (cf. Section 3.2) as, in this experiment, the dwell time is held constant and rather small (1 s), hence leaving less time for ink reflow to the tip apex [21]. Compared to reported feature ranges in PPL with thiols [7], the observed feature range in PPL with phospholipids is smaller, probably due to the bigger role of surface spreading and lipid reorganization here [16].

### 3.6. Demonstration Experiments for Biological Applications

As a demonstration of feasibility to use the obtained lipid structures for biological experiments, we chose the presentation of a model allergen, dinitrophenol (DNP), to mast cells. This system was previously studied for patterning via photolithography [28] and in DPN-generated [29,30,31] lipid structures and could therefore act as an ideal benchmark for our purposes. In particular, the larger surface area of pattern coverage easily achieved by PPL in a timely manner opens up the route for more controlled, parallel experiments by introducing microfluidic chambers, enabling a chip format for mast cell experiments [32].

**Figure 7:**
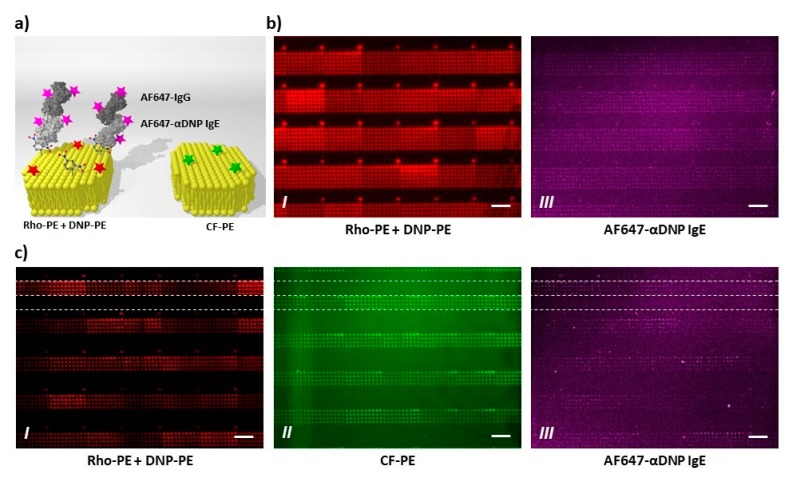
Specific antibody binding on antigen-containing lipid arrays. (**a**) Schematic outline of the performed experiments. The presented antigens were specifically stained by Alexa Fluor^®^ 647 (AF647)-labeled anti-dinitrophenol (DNP) IgE antibodies, followed by incubation with an AF647-labeled secondary antibody. AF647 signal from the labeled antibodies is expected in co-localization with spots containing DNP-conjugated lipids (DNP-PE, left part of scheme), while no signal should be present in absence of DNP-PE (right); (**b**) One-color experiments: 6 × 10 spot arrays containing DOPC (as carrier lipid), dotted with 1% Rho-PE and with 10% DNP-conjugated lipid (DNP-PE), were printed; (**c**) Two-color experiments: Two adjacent 4 × 10 spot arrays were printed with two different inks. The first contains DOPC (as carrier lipid), 1% Rho-PE, and with 10% DNP-conjugated lipid (DNP-PE), while the second contains DOPC and 2% Carboxyfluorescein-conjugated lipids (CF-PE). The results confirm accessibility and specific recognition of the presented antigens, since AF647 signal (panel III) is detected in co-localization with DNP-containing arrays (panel I) and absent on negative control arrays (panel II). Note that the number of columns and rows in the arrays appear inverted, since the images were clockwise rotated for a clearer results interpretation of two-color experiments. In addition, the images in Figure S9 ensure no crosstalk between the fluorescence channels, confirming that the detected AF647 signal uniquely rises from bound antibodies. All scale bars equal 50 µm.

As first test of bioactivity, the recognition of DNP-bearing lipid membrane features by anti-DNP antibodies (ABs) was trialed (Figure 7). First, patterns of 6 × 10 spot arrays containing DNP-PE and admixing of fluorescent Rho-PE for identification were printed via PPL. These patterns, when exposed to fluorescently labelled anti-DNP ABs, show a nice co-localization of pattern and bound anti-DNP ABs (Figure 7b). As the multiplexing capabilities of PPL offer the opportunity for an intrinsic negative control, a second layout consisting of alternating lines of 6 × 10 spot arrays either containing Rho-PE and DNP-PE or CF-PE as admixing to DOPC was printed and incubated with the labelled anti-DNP AB. Again, a nice co-localization of the bound anti-DNP AB is found, while the background and lipid features without DNP-PE admixing remain free from anti-DNP AB adhesion (Figure 7c).

**Figure 8:**
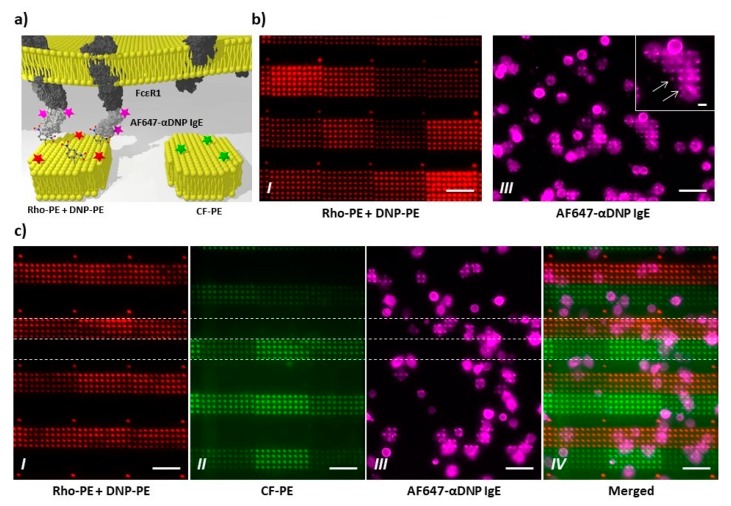
Recruiting of cells on antigen-containing lipid arrays. (**a**) Schematic outline of the performed experiments: RBL2H3 cells were pre-incubated with AF647-labeled anti-DNP IgE antibodies. The excess of antibody was washed away, and the cells were incubated onto previously printed and washed DNP-containing lipid arrays; (**b**) Preliminary experiments to confirm the presence of the antigen DNP in the printed arrays and their accessibility to the specific IgE antibodies; (**c**) Results of the carried experiments. Note that the cells react to the DNP-containing spots by localized spots recognition. In fact, some spots co-localize with region, where the cells accumulate the receptors, indicated by white arrows. All scale bars equal 50 µm.

Next, the biological activity of the PPL-printed allergen patterns was trialed with live mast cells from rat (Figure 8). Again, single ink patterns containing DNP-PE and Rho-PE admixed to DOPC were printed. These patterns were then incubated with mast cells sensitized with fluorescently labelled anti-DNP IgE AB. Only cells landing on an allergen featuring lipid feature show co-localization of their receptor-AB complex with the underlying lipid features (Figure 8b). This co-localization of receptor-AB complex with the DNP features shows the accessibility of the allergen for recognition by the suitable sensitized mast cell and can act as proxy for mast cell activation [29]. To further trial the mast cell response on the PPL-printed patterns, patterns of alternating arrays of DNP-bearing and non-allergen-bearing lipid features were printed and again incubated with sensitized mast cells (Figure 8c). As expected, the cells only show receptor clustering on the DNP-bearing lipid arrays, while cells lying on the non-allergen presenting lipid arrays exhibit no clustering of receptor. The results of this section show the bioactivity of the PPL-printed lipid features and their feasibility for application in cell studies.

## 4. Conclusions

Polymer pen lithography with phospholipids can generate large area arrays of lipid features of varying size. While the general trends in regard to feature size result in regard to control parameters, such as dwell time, ambient humidity, and printing pressure applied to the stamp, hold true in comparison to PPL printing with other materials, the dependence of dwell time and printing pressure was observed to be less pronounced in PPL with phospholipids. Our results shed a first light on the parameters influencing the printing results in PPL with phospholipids and the similarities and differences to PPL with other ink types. For further homogenization of printing results (reducing tip to tip variance), special emphasis should therefore be laid on inking processes to ensure homogeneous coating. Additionally, our results suggest that ink depletion effects can be avoided by printing at raised humidity (>40%). The resulting phospholipid arrays are stable upon immersion in aqueous media and can be used for biological experiments with cells, as exemplified by presentation of a model allergen to mast cells in a lipid array. These structures open up many diverse applications in such experiments due to their antifouling and biocompatible properties, while being open to (bio)functional admixing, e.g., for the generation of functional and heterogeneous supported lipid bilayers [2].

## Supplementary Materials

The following are available online at https://www.mdpi.com/2073-4360/11/5/891/s1, Figure S1: Representative microscope images of the polydimethylsiloxane PDMS stamp obtained by the microscope coupled to the printer setup, Figure S2: Results of the benchmarking of the environmental control system. Figure S3: Alignment challenge and offset correction in two-color printing, Figure S4: Results and analysis of two-color printing experiments, Figure S5: Intensity/area vs. dwell time plots without normalization, Figure S6: Comparison of six independent experiments to analyze the influence of dwell time on lipid ink transfer, Figure S7 Intensity/area vs. relative humidity plots without normalization along with recorded images, Figure S8: Plot of the average fluorescence intensities against Δd, Figure S9: Analysis of signal crosstalk between the different fluorescence channels.
