# Supplementary material for: Writing Behavior of Phospholipids in Polymer Pen Lithography (PPL) for Bioactive Micropatterns
Source: Polymers (Basel). 2019 May 15;11(5):891. doi: 10.3390/polym11050891 (PMC6572014; doi:10.3390/polym11050891)
Supplement: Supplementary file 1 [file polymers-11-00891-s001.pdf]

polymers

Supporting information for

Writing behavior of Phospholipids in Polymer Pen Lithography (PPL) for Bioactive Micropatterns

Alessandro Angelin [1], Uwe Bog [2], Ravi Kumar [2], Christof M. Niemeyer [1] and Michael Hirtz [2,*]

1 Institute of Biological Interfaces (IBG-1), Karlsruhe Institute of Technology (KIT), Hermann-von-Helmholtz-Platz 1, 76344 Eggenstein-Leopoldshafen, Germany
2 Institute of Nanotechnology (INT) & Karlsruhe Nano Micro Facility (KNMF), Karlsruhe Institute of Technology (KIT), Hermann-von-Helmholtz-Platz 1, 76344 Eggenstein-Leopoldshafen, Germany
* Correspondence: michael.hirtz@kit.edu; Tel.: +49-721-60826373

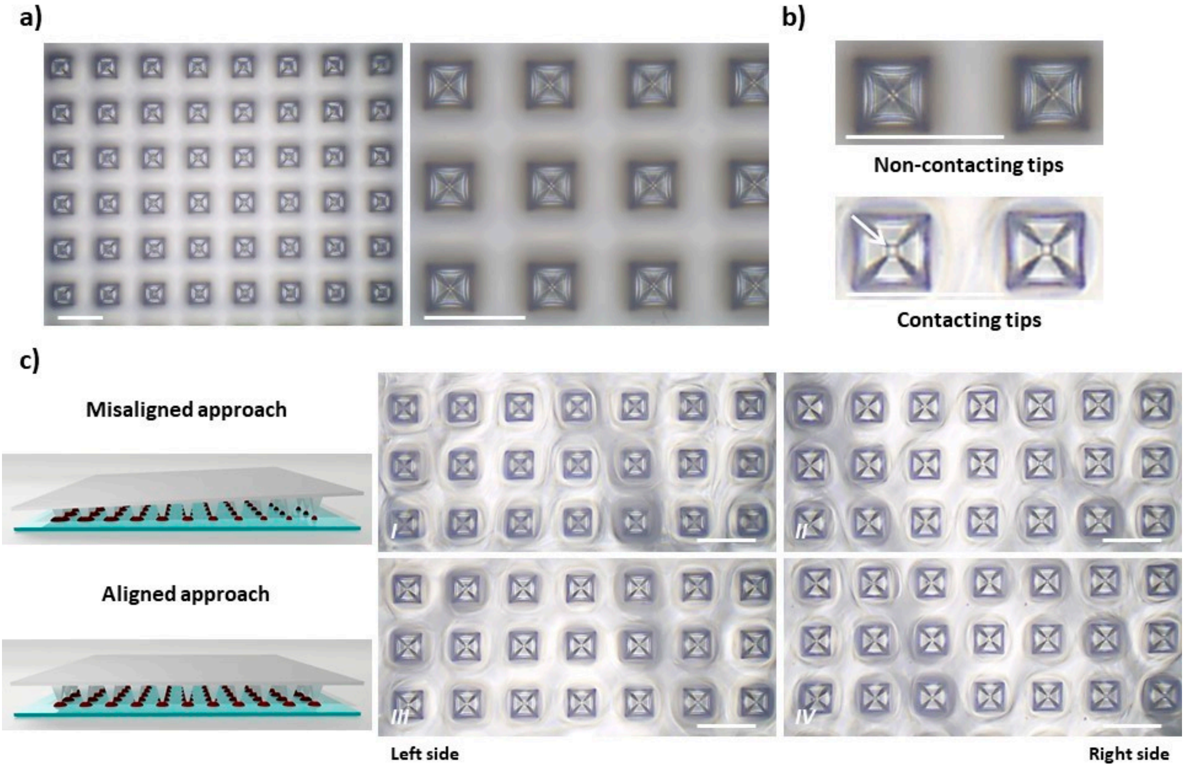

Figure S1. Representative microscope images of the polydimethylsiloxane (PDMS) stamp obtained by the microscope coupled to the printer setup. (**a**) Low (left) and high (right) magnification of the pyramid-containing stamp. (**b**) Representative images of non-contacting (top) and contacting (bottom) pyramids. Note that the elastic deformation of the pyramid's tip induced by the contact can be revealed by the appearance of a distinctive squared area around the center of the pyramid (indicated by the white arrow). (**c**) Representative images obtained after the approach between tips and substrate in case of misaligned (top) and aligned (bottom) planes. A contact gradient can be observed from right to left in panels I and II. After correct plane alignment, an equal contact pressure can be observed for all pyramids in panels III and IV. The scale bars equal 100 µm.

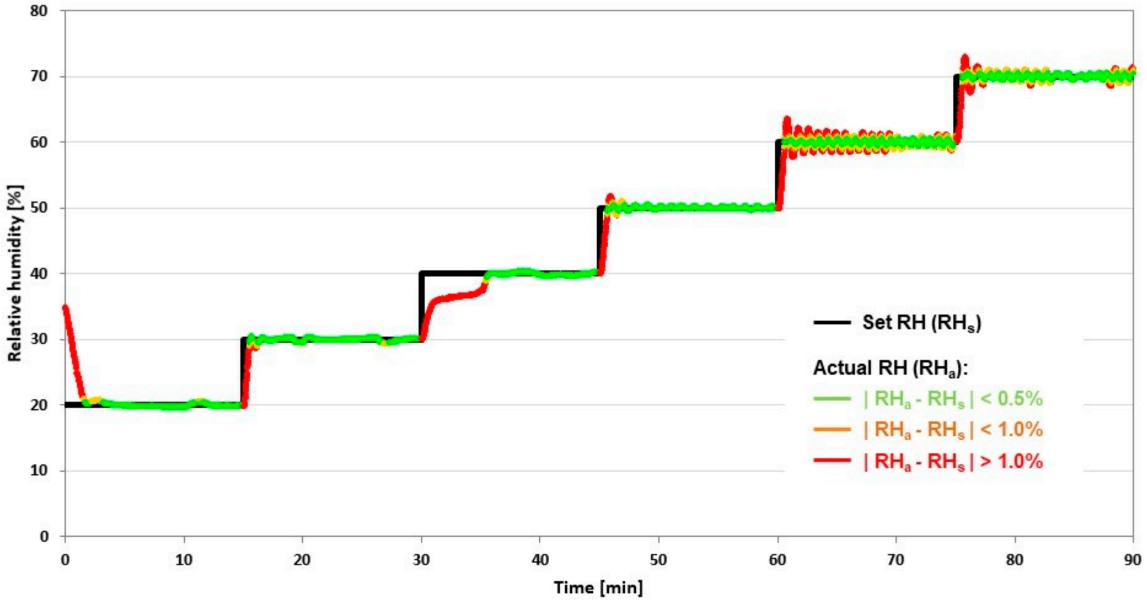

Figure S2. Results of the benchmarking of the environmental control system. The black line indicates the set relative humidity (RH_s), while the color-coded line shows the actual relative humidity (RH_a) measured inside the plastic chamber. Green line: $|RH_a\text{-}RH_s| < 0.5\%$. Orange line: $|RH_a\text{-}RH_s| < 1.0\%$. Red line: $|RH_a\text{-}RH_s| > 1.0\%$. The results indicate that the environmental control works generally within a remarkable range of ±0.5% for RH_s up to 50%. Above this threshold, a slightly increased oscillation in the values is evident, although mainly within a range of ±1.0%, which is still acceptable for such a control system.

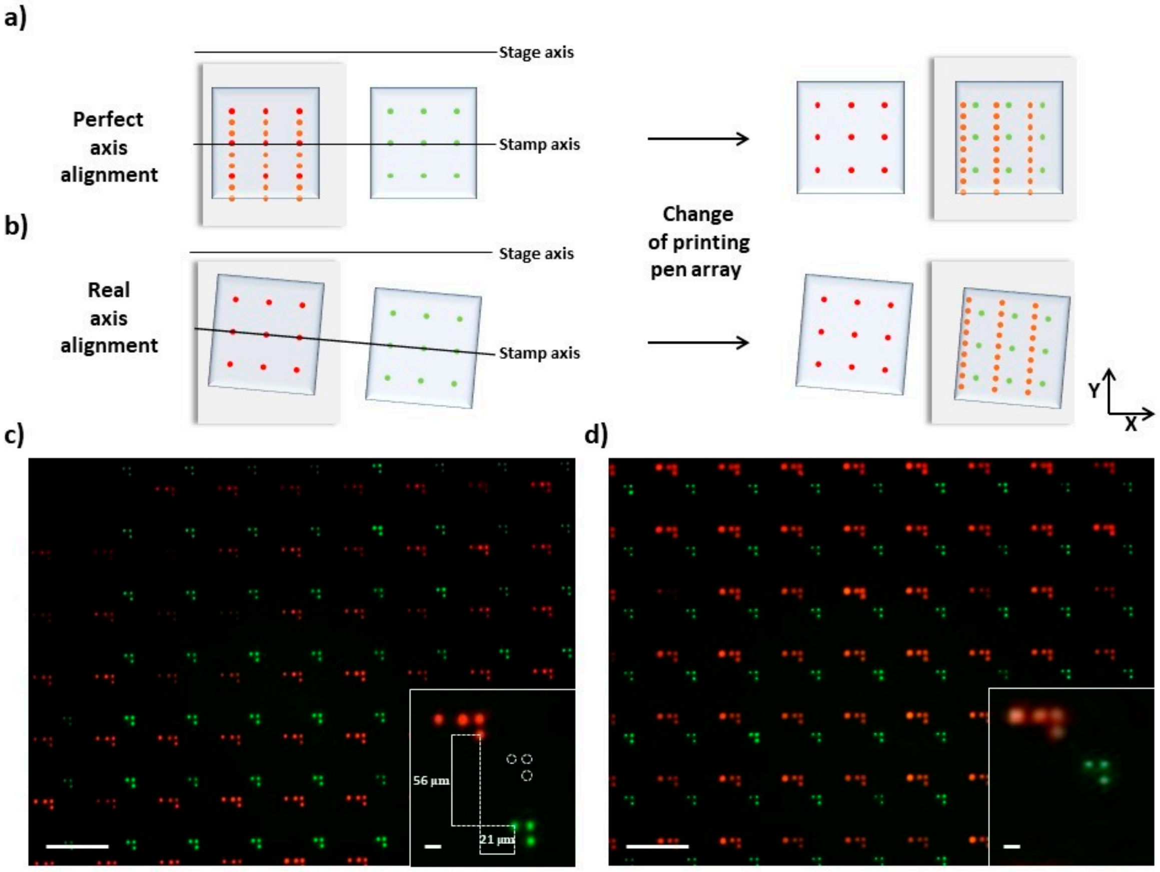

Figure S3. Alignment challenge and offset correction in two-color printing. A schematic representation of the printing of two colors in case of a perfect axis alignment (**a**) and in the real axis alignment situation (**b**). The fluorescence images of a ″three-spot″ test before (**c**) and after (**d**) the offset correction show the effectiveness of the chosen compensation method. The two printed inks are composed by DOPC, which was separately dotted with 1% Rho-PE (in red) and with 2% CF-PE (in green). Higher magnification images of a single two-color array are shown in the insets. In the inset of panel (**c**)**,** the expected positions of the green spots are indicated as dashed circles. The scale bars equal 100 µm in the main images and 10 µm in the insets.

a)

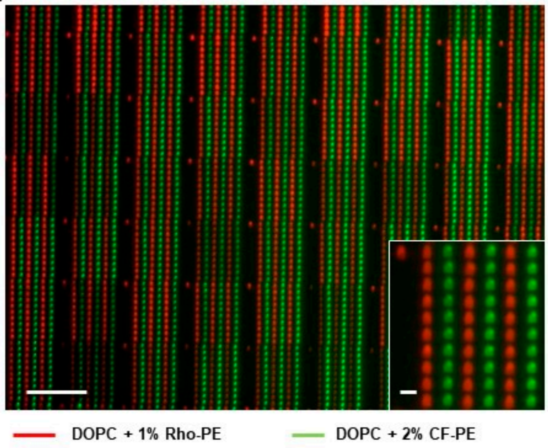

b)

Distance	Measured (mean±SD) [µm]	Theoretical [µm]
A	9.8±0.5	10
B	9.9±0.4	10
C	24.6±0.6	25
D	24.8±0.6	25
E	12.8±0.6	12.5
X Offset 1	0.1±0.4	0
X Offset 2	0.1±0.4	0
Y Offset 1	0.1±0.5	0
Y Offset 2	0.2±0.5	0
Y Offset IC	0.5±0.8	0

c)

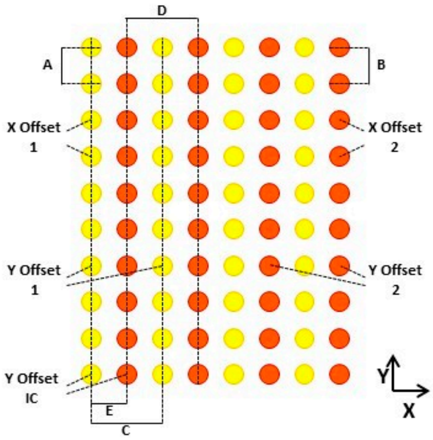

Figure S4. Results and analysis of two-color printing experiments. (**a**) Two different lipids inks, containing DOPC doped with 1% Rho-PE (in red) and with 2% CF-PE (in green), were printed on a cleaned bare glass substrate. Each color was used to print a 10 × 4 pattern with 20 µm pitch, and the stamp was aligned to obtain the two patterns superimposed with a pitch of 12.5 µm. The scale bars equal 100 µm in the main images and 10 µm in the inset. (**b**) Results of the analysis of relative distances between neighboring spots in 15 different arrays from the image in panel (**a**). The measured distances were named according to the scheme reported in (**c**). The alignment of two consecutive spots (within the same color pattern) along the x and y axes are indicated as X- and Y-offsets n, respectively, where n indicates the ″color number″. The offset between the two colors is indicated as Y Offset IC (inter-color). Note that the measured distances are in accordance with the theoretical values, confirming the success of alignment and printing procedure. The pitches between neighboring spots were calculated from the spots′ coordinates via the Pythagorean theorem. First, the analyzed micrographs were rotated to compensate for the tilted camera during image acquisition. To this aim, the amplitude of necessary rotation was determined, taking as reference two distant corresponding spots (same row and column from distant pyramids). Finally, the differences between the absolute coordinates of spots were used to determine the distances reported in the table.

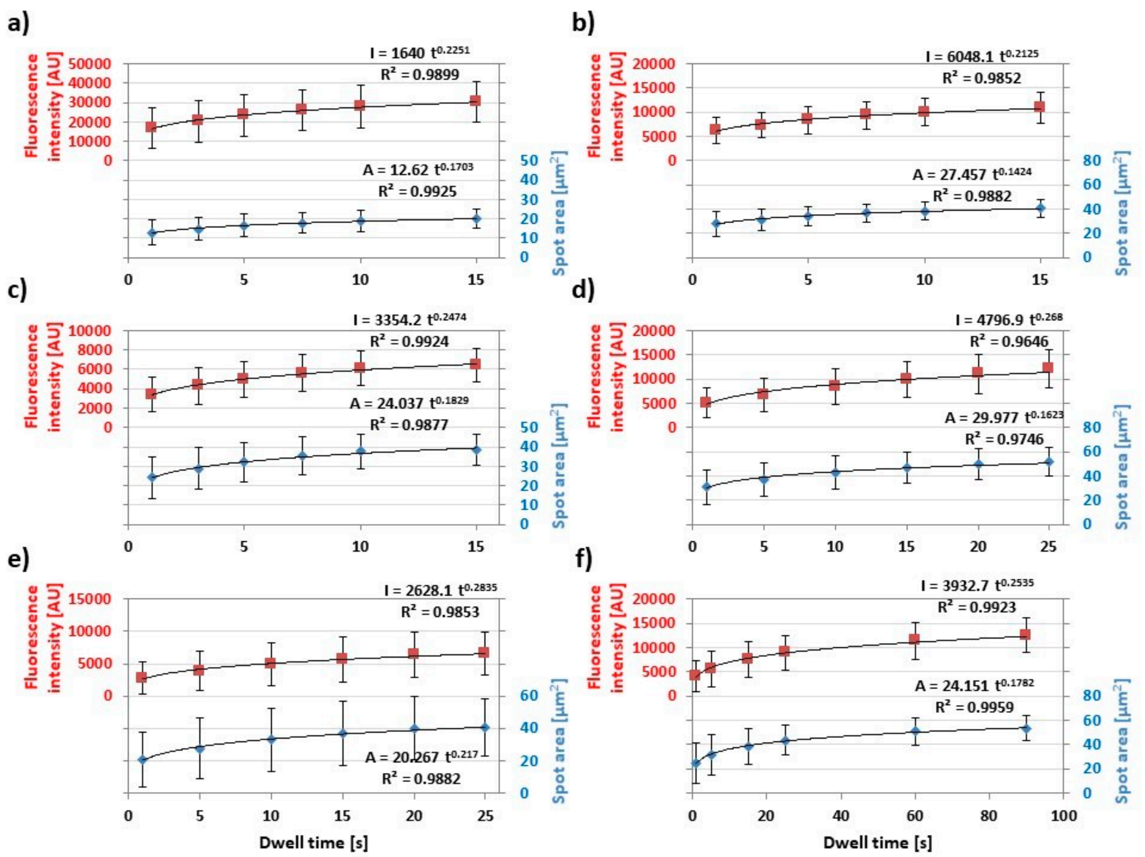

Figure S5. Intensity/area vs. dwell time plots without normalization. Plots of the average intensity (red) and area (blue) of spots against dwell time for six independent experiments. For each slide, 30 arrays were analyzed. The substrates in panels (**b**) to (**f**) were sequentially printed using the same stamp. Different dwell time ranges were analyzed in the different experiments: 1 to 15 seconds in panels (**a**), (**b**) and (**c**), 1 to 25 seconds in panels (**d**) and (**e**), 1 to 90 seconds in panel (**f**). All the printings were performed under 40% RH and room temperature. Error bars represent standard deviation.

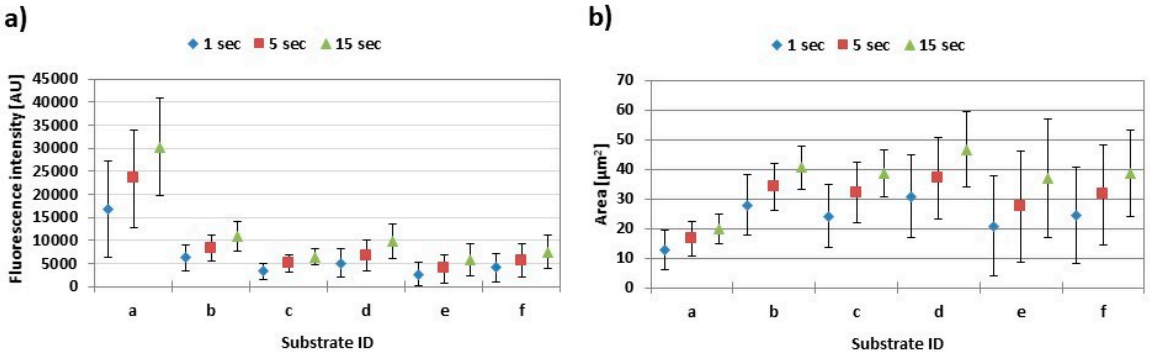

Figure S6. Comparison of six independent experiments to analyze the influence of dwell time on lipid ink transfer. The average fluorescence intensity (**a**) and area (**b**) in the six experiments are plotted for the dwell times of 1, 5 and 15 seconds. The x axis reports the substrate IDs that correspond with the panel letters of Figure S5 of the main manuscript. Note that the data points are not plotted with numerical order on the x axis but rather with category labels. This implies that the shown linear behavior is only apparent. For the real data trend and discussion, refer to Figure 3 and to the related text section. Error bars represent standard deviation.

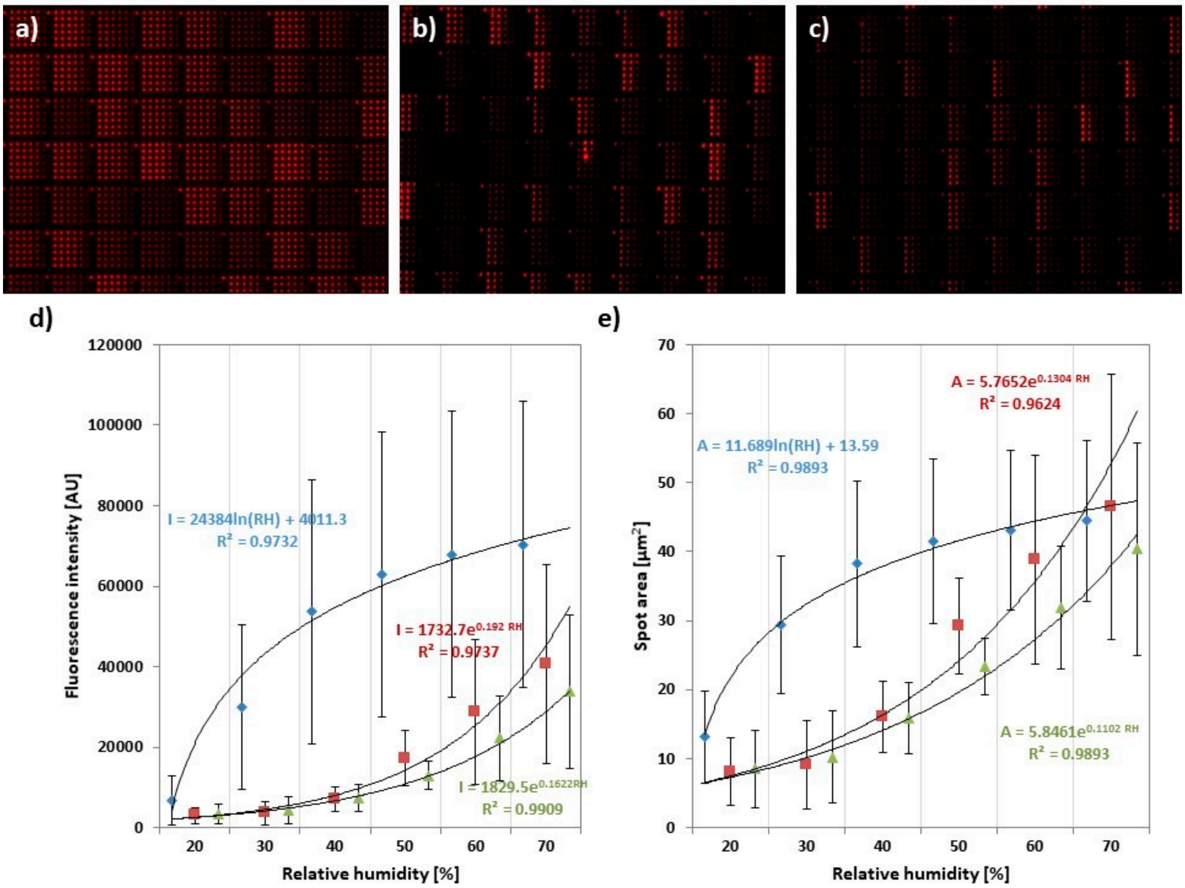

Figure S7. Intensity/area vs. relative humidity plots without normalization along with recorded images. (**a**–**c**) Fluorescence micrographs of three independent experiments carried out to test the influence of humidity on the amount of transferred ink. The images of these three independent experiments were analyzed by ImageJ, as reported in the plots of the average intensity and area of spots against relative humidity (**d**–**e**). In the graphs, the blue, red and green curves correspond to the images in panels (**a**), (**b**) and (**c**), respectively. For each slide, 30 arrays were analyzed. Error bars represent standard deviation.

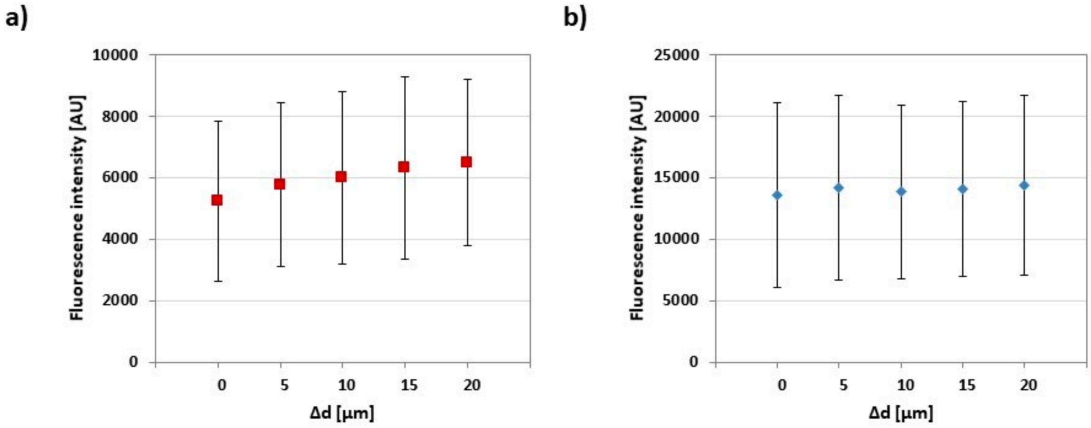

Figure S8. Plot of the average fluorescence intensities against Δd in two independent experiments presented in Figure 6. Error bars represent standard deviation.

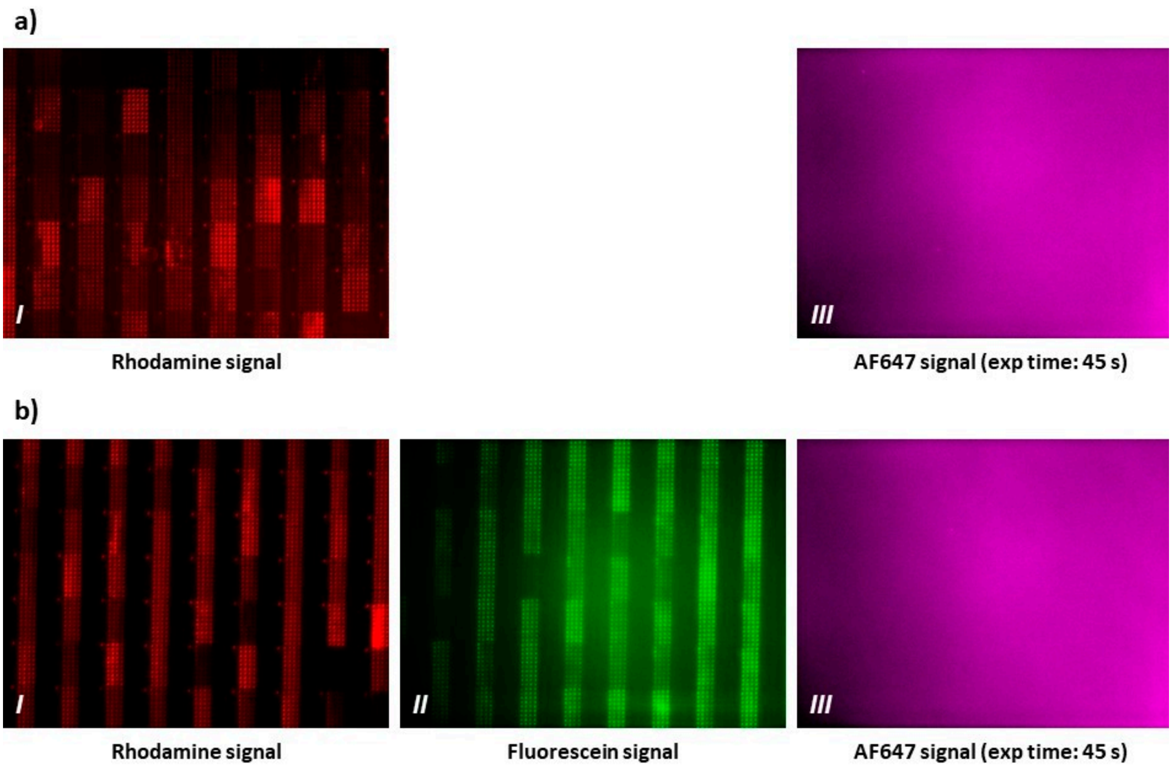

Figure S9. Analysis of signal crosstalk between the different fluorescence channels. Importantly for the interpretation of the results in Figure 7 of the main manuscript, no appreciable signal from the spots is shown by the AF647 (Alexa Fluor® 647) channel even at the maximum exposure time of 45 seconds.
